# Effects of a multi-strain probiotic on productive traits, antioxidant defence, caecal microbiota and short-chain fatty acid profile, and intestinal histomorphology in rabbits

**DOI:** 10.5713/ab.24.0716

**Published:** 2025-01-24

**Authors:** Vincenzo Tufarelli, Caterina Losacco, Gianluca Pugliese, Alessandra Tateo, Michele Schiavitto, Fabrizio Iarussi, Vito Laudadio, Letizia Passantino

**Affiliations:** 1 Department of Precision and Regenerative Medicine and Jonian Area, Section of Veterinary Science and Animal Production, University of Bari Aldo Moro, Valenzano, Bari, Italy; 2 Italian Rabbit Breeders Association (ANCI-AIA), Volturara Appula, Foggia, Italy

**Keywords:** Caecal Environment, Gut Morphology, Microbiota, Probiotic, Rabbit

## Abstract

**Objective:**

This paper aimed to evaluate the effects of a multi-strain probiotic (MS-Prob) as natural feed additives on the productive performance, blood parameters, antioxidant defence, caecal short-chain fatty acid profile as well as the effectiveness on the intestinal morphology and on the equilibrium modification of caecal microbiota of growing rabbits.

**Methods:**

Eighty-six-week-old Italian White rabbits were assigned into two dietary groups: the control group was fed without any additive, while the test group received a diet supplemented with a MS-Prob (Slab51) at a dosage of 250 mg/kg diet. The feeding trial lasted up to 12 weeks of age.

**Results:**

Rabbits MS-Prob diet had significantly higher daily and final body weight recording also the best of feed efficiency compared to control group. Rabbits in MS-Prob group showed improved slaughter weight and carcass dressing yield. No significant effect was found on meat protein, lipids and ash contents. Serum total cholesterol, low-density lipoprotein, high-density lipoprotein and triglycerides decreased significantly in rabbits fed the test-diet. In rabbits fed MS-Prob, the activity of glutathione peroxidase, glutathione S-transferase, catalase and superoxide dismutase increased significantly, whereas the level of thiobarbituric acid-reactive substance decreased significantly. Caecal pH, ammonia-N and total volatile fatty acids (VFAs) were not significantly influenced by dietary treatments. Conversely, VFAs molar proportions were statistically affected by diets, with higher acetic and butyric acid concentrations in caecum of rabbits fed MS-Prob. Feeding of MS-Prob reduced harmful bacterial growth (*Escherichia coli*, *Bacillus* spp., *Clostridium* spp.) while promoting beneficial bacteria (*Lactobacillus* spp., *Bacteroides* spp.). Moreover, MS-Prob positively influences rabbit gut histomorphology, showing higher villus height, villus width, and crypt depth.

**Conclusion:**

This study indicated that MS-Prob (Slab51) supplementation stimulated the performance of growing rabbits and positively affected blood serum parameters, intestinal morphology, and caecal environment and microbiota.

## INTRODUCTION

Due to their ability to promote growth in livestock species, natural feed additives such as probiotics are a viable substitute for drugs and antibiotics [1]. Probiotics are bio-preparations that enhance the composition and colonization of gut microflora in both humans and animals and have a stimulatory influence on digestive processes. They include live metabolites or cells of stable autochthonous bacteria [2]. Probiotics work mainly by competing with pathogenic bacteria for the intestinal mucosa’s attachment sites, preventing their adhesion by creating a physical barrier, and by stimulating the immune cells and epithelial cells activity [3]. As a result, adding natural additives to an animal diet can improve its health and productivity, including rabbit [4]. According to study findings, adding probiotics to the feed of livestock species also reduced mortality and enhanced growth [5].

Rabbit meat consumption and production are important in Mediterranean nations, notably in Italy, France, and Spain, therefore enhancing rabbit health and meat quality may encourage consumers to use this alternative meat source [6]. However, rabbits’ high feed conversion ratio (FCR) leads to poor yield and high breeding costs; hence, different feeding techniques are being investigated to increase performance, health, and meat output [7]. Recently, it has been shown that the dietary supplementation with a commercial multi-strain probiotic (MS-Prob; Slab51; Ormendes SA, Jouxtens-Mézery, Switzerland) containing a mixture of different species of lactic acid bacteria and bifidobacteria induced enhancement of growth performance and improvements in gut morphology of animals [1]. *Lactobacillus acidophilus*, in particular, has been shown to reduce bacterial cell proliferation, migration, and invasion potential. Moreover, several studies have shown that *Lactobacillus* spp. has the ability to regulate the diversity and activity of the intestinal microbiota, leading to better intestinal health and epithelial function [8].

Thus, probiotics offer a viable and long-term substitute for improving rabbit health and productivity while lowering the reliance for antibiotics [9]. Furthermore, their potential to optimize animal health and productivity is highlighted by their capacity to support immunological function, optimize food consumption, inhibit the emergence of potentially hazardous infections, and maintain a healthy gut microbiota [8].

Therefore, the aim of this study was to evaluate the effects of a MS-Prob as natural feed additives on the productive performance, blood parameters, antioxidant defence, caecal short-chain fatty acid profile as well as the equilibrium modification of the caecal microbiota population of rabbits.

## MATERIALS AND METHODS

### Animals, management and feeding

Rabbits in the present study were cared and handled in compliance with the EU legislation on animal welfare regulations (Directive 2010/63/EU, which updates and replaces the 1986 Directive 86/609/EEC) and following the research policies of the DiMePRE-J of the University of Bari Aldo Moro, Italy (Approval code 09/2022). The research was conducted at the rabbitry of the Genetic Center of the Italian Rabbit Breeders Association (ANCI-AIA; Volturara Appula, Italy). Eighty male Italian White (Bianca Italiana) growing rabbits, and aged 42 days (body weight 1,095±29.3 g, mean±standard error of the mean [SEM]), were randomly assigned to two groups of 40 animals according to the dietary treatment. The feeding trial lasted up to 84 days of age (12 weeks). The control group was fed without any additive, while the test group received a diet supplemented with a MS-Probmixture Slab51 at a dosage of 250 mg/kg diet. Slab51 (marketed in Europe under the trademark SivoMixx, Ormendes SA) is a commercial MS-Prob containing 200 billion lactic acid bacteria per 1.5 g of product, comprised of the following strains: *Streptococcus thermophilus* DSM 32245/CNCM I-5570, *Bifidobacterium lactis* DSM 32246/CNCM I-5571, *Bifidobacterium lactis* DSM 32247/CNCMI-5572, *Lactobacillus acidophilus* DSM 32241/CNCM I-5567, *Lactobacillus helveticus* DSM32242/CNCMI-5573, *Lactobacillus paracasei* DSM 32243/CNCM I-5568, *Lactobacillus plantarum* DSM 32244/CNCM I-5569, and *Lactobacillus brevis* DSM 27961/CNCM I-5566. The rabbits were housed as four rabbits per cage in galvanized wire single cages within open-system pens (35×40×50 cm) in width, height, and length (Italian battery) and at a height of 90 cm from the concrete floor. Hand feeding and an automated nipple drinker system offered constant access to fresh, clean water for every cage. The rabbits were allotted under identical environmental and sanitary conditions throughout the trial. The ingredients composition and chemical analysis of diet are shown in Table 1. No medication was included in the feed or in the drinking water and rabbits’ health status was checked through individual observations.

### Growth, carcass traits and meat quality

From 6 to 12 weeks of age, rabbits were weighed individually at weekly intervals, while the feed intake was daily recorded and the FCR was calculated. Health status, clinical illness and mortality were monitored daily. Mortality rate was considered when calculating the FCR. At the end of the fattening period at 84 days of age, 10 rabbits per group were randomly selected in the afternoon for slaughter. On the next morning, the selected rabbits were transferred in small groups to the slaughter facility near the rearing building in the slaughter to determine carcass traits. The rabbits were then weighed, electrically stunned, and slaughtered within 2 h. The slaughtering and carcass dissection procedures followed the World Rabbit Science Association recommendations [10]. The rabbits were bled, and then the skin, gastrointestinal tract and the distal part of legs were removed. Carcasses (with head, thoracic cage organs, liver, kidneys, perirenal and scapular fat) were weighed (hot carcass), then chilled at 4°C for 24 h in a ventilated room. After chilling, the chilled carcasses were weighed and the slaughter yield calculated. Samples of meat from the *longissimus lumborum* muscle were stored at −80°C for assessing lipids content, and others were individually stored in plastic bags at 4°C for meat quality analysis. Meat samples were analysed in triplicate for dry matter by oven drying method (934.01), total ash by muffle furnace (942.05) and protein by Kjeldahl method (954.01) as following the AOAC methods. Total lipids were extracted according to the method of Folch et al [11].

### Blood serum lipids and antioxidant capacity analysis

Ten blood samples from each treatment were taken from the rabbit marginal ear veins at the end of the trial (84 days of age), one hour before the regular feeding time. After the samples were gathered into sterile tubes and centrifuged, the serum was separated and stored refrigerated (−20°C) for further examination. The obtained serum samples were subjected to measurements of the following parameters: triglyceride (TG; mg/dL), high-density lipoprotein (HDL; mg/dL), low-density lipoprotein (LDL; mg/dL), and total cholesterol (TC; mg/dL). Using commercial kits, antioxidant activities including thiobarbituric acid-reactive substances (TBARS), glutathione peroxidase (GPx), glutathione S-transferase (GST), catalase (CAT), and superoxide dismutase (SOD) were measured in accordance with the manufacturer’s instructions [12].

### Caecal characteristics and microbiota

Volatile fatty acids (VFAs) were assessed in the caecal contents of slaughtered rabbits using gas chromatography, as described by Abu Hafsa et al [13], and ammonia was analyzed using an automated distillation unit. The pH of caecal contents was also determined using a portable pH meter. Then, the caecal content was collected into sterile plastic tubes and frozen at −80°C for microbiota analyses. Bacterial counts were conducted on the same caecal material to determine the main bacterial species present in rabbits. Aerobic and facultative anaerobic bacterial counts on tryptic glucose yeast agar, Enterobacteriaceae and coliforms on violet-red bile glucose agar, *Escherichia c*oli on chromogenic coliform agar, *Lactobacillus* spp. on De Man-Rogosa-Sharpe agar, and *Clostridium* spp., *Bacteroides* spp., and *Bacillus* spp. on phenylethyl alcohol agar, were determined according to Maturin and Peeler [14]. Lastly, bacterial colonies on plates were counted with an optical counter.

### Histomorphometric examination

At the day 84, all the slaughtered rabbits were submitted to histomorphological and morphometric evaluation. Samples of duodenum (at 3 cm from the pylorus) and approximately 2 cm in length were excised and flushed with 0.9% saline to remove the contents. To the specimen were applied the routine histological methods; briefly, segments were fixed in 10% neutral-buffered formalin solution then dehydrated, cleared, and paraffin embedded. Intestinal samples were trimmed and transverse sections of 5 to 7 μm were placed on glass slides and stained with hematoxylin and eosin (H&E) (Merck, Darmstadt, Germany) for morphological and morphometric studies and Azan Mallory trichrome stain (Merck) to distinguish cells from the extracellular matrix and to highlight collagen fibres in connective tissue. The presence of goblet cells (GCs) was demonstrated by periodic acid-Schiff (PAS) staining sequence, which reveals acidic, neutral, and mixed mucins. The PAS (Merck) detects the presence of glycogen, polysaccharides, and mucin in the tissue through a reaction with carbohydrates in an oxidative process, which, ultimately, stains the samples with a purple-magenta color, similarly, the appearance of the pink color suggests the presence of intracellular or extracellular mucin.

For histomorphometric evaluations, H&E stained sections of 20 well-oriented villi and 30 crypts of the duodenum were selected from each intestinal cross-section. The morphometric indices evaluated were: villus height (VH) from the tip of the villus to the crypt and crypt depth (CD) from the base of the villi to the submucosa, then the ratio of the villus height to crypt depth (VH:CD) was calculated [15]. The apparent villus surface area was calculated by the following formula: [(villus width at one-third+villus width at two-thirds of the height of the villus)×2^−1^×VH], according to Tufarelli et al [16]. All morphological measurements (VH and CD) were evaluated at 10× and 25× magnification by using an image analysis system (X-Series, Alexasoft, Firenze, Italy).

### Statistical analysis

Data were analyzed as one-way ANOVA design, using the GLM procedure of SAS (version 9.2; SAS Institute, Cary, NC, USA). Means were separated and compared by Tukey’s honestly significant difference test. Results were reported as least squares mean and pooled SEM. Statistical significance was considered at p≤0.05.

## RESULTS

### *In vivo* performance and meat traits

Results indicated that the MS-Prob inclusion had a significant effect on the growth performance of rabbits (Table 2). Indeed, the test group registered a higher final body weight (p = 0.004) and gain (p = 0.003) than the control. Feed intake did not differ (p>0.05) between groups; however, the FCR was improved by MS-Prob (p<0.001) and mortality was significantly reduced by dietary probiotic (p<0.001) compared to control. Dietary supplementation with MS-Prob had an effect on rabbit carcass traits (Table 2). Slaughter weight, hot carcass weight and carcass yield increased significantly (p<0.05) for rabbits fed MS-Prob compared to those in control diet. Meat from *Longissimus lumborum* muscle did not differ (p>0.05) between groups in terms of moisture, protein, lipids and ash contents.

### Blood lipids and antioxidant activity

Serum TC, LDL, HDL and TG decreased significantly (p< 0.005) in rabbits fed MS-Prob compared to the control diet (Table 3). Regarding the oxidative status of investigated rabbits, the findings indicated that GPx, GST, CAT and SOD increased significantly (p<0.005), whereas TBARS decreased significantly (p = 0.001) with the dietary inclusion of the MS-Prob compared to the control group.

### Caecal fermentation profile and microflora

The caecal environmental characteristics and the microflora population in rabbits at 12 weeks days of age are reported in Table 4. The values of caecal pH, ammonia-N and total VFAs were not significantly influenced by dietary treatments. Furthermore, VFA molar proportions were statistically affected by the diets, with higher acetic and butyric acid concentrations in caecum of rabbits fed MS-Prob (p<0.05); whereas, propionic acid and valeric acid concentrations did not differ between groups (p>0.05).

Results from caecal microbiota analysis showed a significant variability between rabbits’ groups. Feeding of MS-Prob significantly decreased caecal *E*. *coli* (p = 0.001), *Bacillus* spp. (p = 0.003), *Clostridium* spp. (p = 0.001) populations, whereas, *Bacterioides* spp. (p = 0.004) and *Lactobacillus* spp. (p = 0.001) populations increased significantly.

### Intestinal histomorphometry

**Figure 1:**
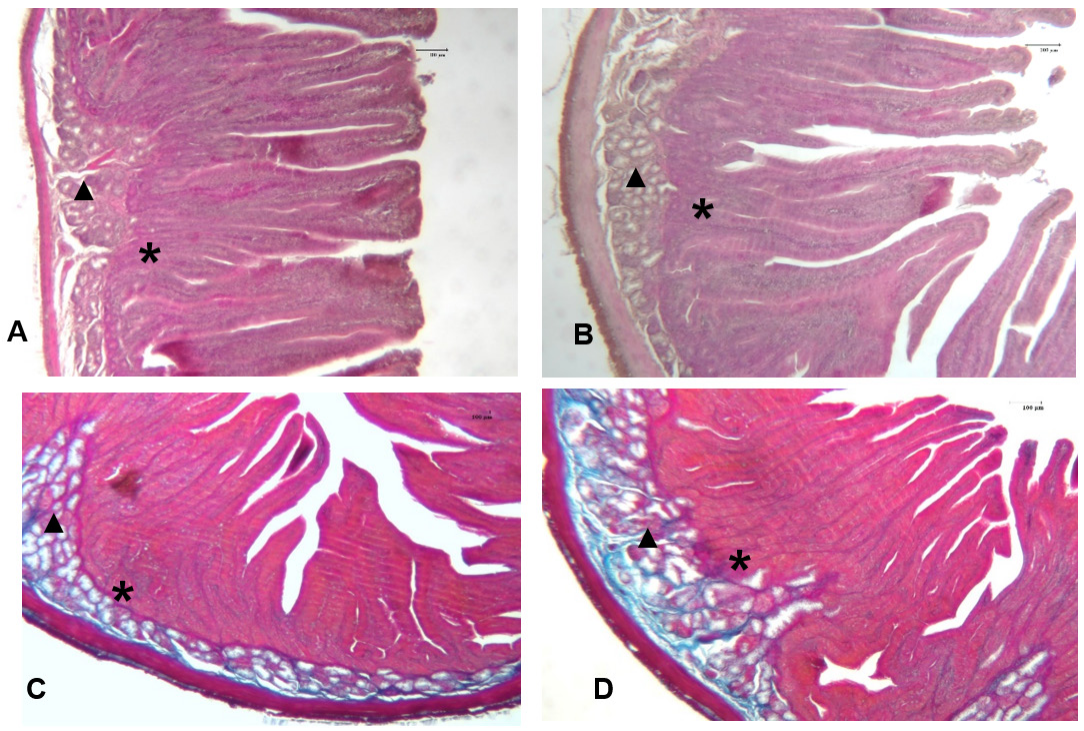
Representative microphotograph of the duodenum of rabbits fed control diet (panel A: H&E and panel C: Azan Mallory, 25×) and multi-strain probiotic (panel B: H&E and panel D: Azan Mallory, 25×). * Crypts of Lieberkühn, ▲ glands of Brunner.

**Figure 2:**
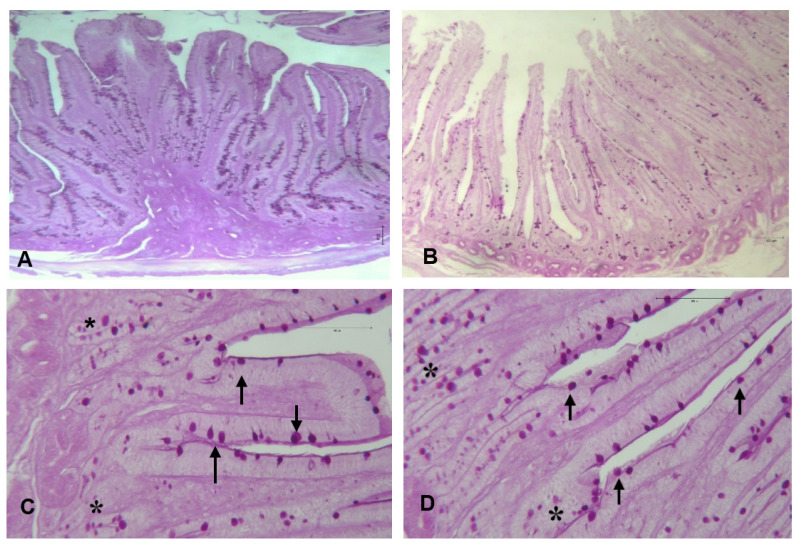
Microscopic image at different magnification of duodenum of rabbits fed control (A: 25× and C: 100×) and multi-strain probiotic (B: 25× and D: 100×) diets stained with Periodic acid-Schiff (PAS). The goblet cells (arrows) and the crypts of Lieberkühn (*) appear in darker pink color.

The effect of the MS-Prob dietary supplementation on the histomorphometric measures and morphologic architecture of the duodenum of rabbits are shown in Table 5 and Figures 1, 2. The VH and the VH:CD were significantly increased (p = 0.001 and p = 0.002, respectively) by feeding probiotic. The morphometric analysis also showed an increase (p = 0.002) of the CD in the experimental group compared to the control. Moreover, dietary supplementation with probiotic resulted in a significant increase (p<0.05) of the villus surface area. The morphological observation of all tested groups displayed a harmonic development of the duodenal mucosa with regular villi shapes that protruded into the lumen. The villi were lined by a layer of absorbent cells, the enterocytes, and GCs distributed among columnar cells. The GCs secrete a protective mucus layer that lines the surface of the epithelium attached on a regular mucosal muscle layer (Figure 1). At the base were visible invaginations of the epithelium surrounding the villi, namely crypts of Liberkuhn (Figures 1C, 1D), these glands together with the GCs resulted positive to PAS stain and appear in darker pink color (Figure 2).

## DISCUSSION

The present feeding trial pointed to evaluate the effects of MS-Prob containing *Streptococcus*, *Bifidobacterium* and *Lactobacillus* strains on growth performance, serum lipid profile, oxidative status and gut health of growing rabbits. Many reports are aimed to identify feed additives that achieve the goal of improve both animals’ production indices and health, facing the effects of exogenous and endogenous stressor factors [17]. In this study, the probiotic mixture exerted a significative effect on growth performance and feed efficiency as well as on carcass traits and mortality rate of the test-group fed probiotic (Table 2). Even if feed intake did not differ between groups, FCR was improved and mortality rate was significantly reduced by dietary probiotic compared to control. Benefits on rabbit health status and mortality rate derived from the use of probiotics were observed in many studies. A recent trial was performed to evaluate the effects on healthy hybrid rabbits of an oral paste containing *Lactobacillus acidophilus* [8]. The rabbits fed probiotic registered an increase in body condition score, while the rabbits in unsupplemented control group showed enteritis and cutaneous abscesses that had worsening the nutritional status of animals. The same Authors referred these results to a reduction of disease occurrence and to a reinforce of the immune status of treated rabbits. In a previous study, Dimova et al [18] recorded a lower mortality rate in both fattening rabbits (11.11%) and weaned rabbits (10,8%) derived from does treated with 0.5% of a probiotic mixture. In the present study, a similar reduction of mortality (10%) was highlighted in rabbits fed MS-Prob that during the trial remained in good health conditions without showing any symptoms of disorders. Our results are aligned with those of Phuoc and Jamikorn [19], who found a significant decrease in morbidity and mortality in rabbits fed a diet supplemented with *Lactobacillus acidophilus*. Also, Abdel-Azeem et al [20] referred a significant reduction in mortality in the trial groups administered with anaerobic probiotic.

Several studies reported that dietary probiotics could raise the *in vivo* performance of growing rabbit. In this trial, rabbits in MS-Prob diet recorded an improved feed efficiency and a significantly higher average daily gain of weight (ADGW) and final body weight compared to control group. Further, MS-Prob group showed improved slaughter weight and carcass dressing yield, while no significant effect was found on meat quality. The obtained results confirm the role of probiotics in enhancing the feed efficiency. It was found that growing rabbits fed a diet supplemented with fenugreek seeds and probiotics recorded a better FCR and a higher digestibility of protein compared to a control group [21]. Recently, El-Sawy et al [22] concluded that using probiotic in drinking water improved productive performance and increased feed utilization without any negative effect on carcass traits in rabbits. Kadjia et al [23] investigated in rabbit the effects of three strains of probiotics: *Lactobacillus rhamnosus*, *Bifidobacterium animalis* subsp. *Lactis*, and *Saccharomyces boulardi* and found that ADGW increased significantly regardless of the used probiotic strain in diet. Under heat stress condition, *Saccharomyces cerevisiae* and *Lactobacillus acidophilus* increased live body weight in rabbit orally administered groups against a control [24]. Similarly, *Lactobacillus acidophilus* or *L*. *lactis* exerted a positive effect on ADGW and FCR in treated rabbits [25]. Improvement in productive parameters of rabbits administered with probiotics are supposed to be induced by the secretion of amylase, protease, and lipase digestive enzymes which in turn promotes nutrient digestibility [19,25] and absorption in the gut [26]. Feeding of *Clostridium butyricum* in weaned rabbit was found to ameliorate weight gain through the probiotic activity on both digestive enzymes and small intestinal morphology [27]. Further, in digestive tract the probiotic complex can multiply and synthetize itself a wide range of digestive enzymes, while utilizing different carbohydrates to produce VFAs, thereby they not only extend the rabbit fermentative digestive system but also have a role in harmonizing the intestinal tract [27,28]. Rabbit microbiota balance contribute to produce positive effects on productive performance and health [9]. Indeed, mounting evidences suggested that probiotics effect on rabbit weight gain and feed utilization could be explained by the influence on gut health and microenvironment that in turn results in enhanced nutrient absorption capacity [28]. Meanwhile, the improvement of intestinal features and nutrients’ availability may be linked to a competitive exclusion of pathogens and a retention of beneficial gut microbiota leading to a reduction of intestinal disorders [19,24].

It is worth noting that in this study the addition of probiotics influenced the carcass traits, while there were no significant variations in meat quality across the groups, as showed in Table 2. Findings on carcass traits and meat quality evaluation reported opposite results in literature. Fathi et al [29] and Mohamed et al [30] reported that dietary probiotic, respectively *Bacillus subtilis* and a mixture of *Bifidobacterium bifidum* with *Lactobacillus acidophilus*, ameliorate most of the carcass traits of tested rabbits, while El-Sawy et al [22] and Hegab et al [24] didn’t find any effect on the carcass characteristics among treatments. Regarding meat quality, significant effects on the proximate composition of meat was found in rabbits fed *Bacillus subtilis* as a probiotic [29], instead Pogáni Simonová et al [31] did not find significant variations in the pH, colour, proximate composition, and water holding capacity in rabbits fed a diet supplemented with *Enterococcus faecium*.

Data presented in this trial showed that MS-Prob affected serum lipid profile of administered rabbits (Table 3). Blood biochemical parameters reported a significant decrease in TC, TG, LDL and HDL, important indicators of lipid metabolism and lipid transport in the body. The improvement in lipid profile has been reported in many investigations on dietary probiotics. A recent study stated that rabbits raised under hot climate conditions, experienced a significant hypolipidemic effect when fed probiotic [32]. Similarly, Khalifa et al [28] reported an improved lipid profile in weaned rabbits supplemented with a probiotic mixture of *Bacillus spp*. The TC and TG levels were found to be significantly decreased in three rabbits’ groups supplemented with three probiotics strains (*Lactobacillus rhamnosus*, *Bifidobacterium animalis* subsp. *Lactis* and *Saccharomyces boulardii*) in a study conducted by Kadja et al [23]. Also, El-Shafei et al [33] found a significant decrease in serum TC and TG in rabbits supplemented with *Lactobacillus plantarum*. It seems that the hypocholesterolemic effect of probiotics could be referred to different pathways. These bioactive complexes may incorporate or bind the intestinal cholesterol into bacterial cells and use it for their cellular metabolism making short fatty acids [34]. Further, studies have shown that some probiotics, like Lactobacilli and Bifidobacteria, are able to hydrolyze bile salts, thereby inhibiting them from working as precursors in cholesterol synthesis [35]. At the same time, these microbial strains may convert cholesterol in coprostanol [36] and inhibit the enzyme involved in the cholesterogenesis, the hydroxyl methylglutaryl-CoA [37].

Regarding rabbit oxidative status, the findings presented in this study indicated that MS-Prob played a positive role on rabbit oxidative markers (Table 3). Compared to the control, the probiotic group showed a remarkable decrease in TBARS levels and a significant increase of the endogenous antioxidant enzymes (GPx, GST, CAT and SOD). Currently, many investigations explored the effects of probiotic intake on animal oxidation and antioxidation markers in order to evaluate their influence on the prevention of oxidative stress damages. Evidences reported that *Lactobacillus* and *Bifidobacterium* could exert antioxidant activities and reduce oxidative damage both *in vivo* and *in vitro* [38]. *Aspergillus awamori* increased the activity of SOD and CAT enzymes and removed the excess of ROS when administered in rabbits with dietary ochratoxin A-induced toxicity, exhibiting a positive influence on the antioxidant resistance of treated group [26]. In weaning rabbits, high-dose of *Clostridium butyricum* increased SOD, GSH-Px, and CAT activities together with a decrease in malondialdehyde content in the intestinal tissues, indicating that probiotic intake enhanced the intestinal antioxidant capacity lastly improving gut health [27].

The establishment of healthy, stable and improved digestive environment is essential for rabbits to resist intestinal disorders, and to maintain health status and productive performance. Dietary probiotic intake was found to exert specific dynamic effects on digestive system via the balance of the microbial environment equilibrium and the modulation of gut morphologic features [34]. On the other hand, it is important to remark that this bioactive mixture may suppress potentially pathogenic microorganisms, like *Clostridium* and coliform, and that MS-Prob appear to be more effective in inhibiting intestinal pathogens than single-strain probiotics, due to the variation in their inhibitory mechanisms of action [39]. Additionally, the high level of diversity promoted by multi-strain bacterial colonization may be considered advantageous due to its potential to reinforce microbiota community resistance to environmental stresses and pathogens [1].

Data on caecal environmental characteristics and microflora composition in this study are reported in Table 4. Dietary treatment did not affect caecal pH, ammonia-N and total VFAs, while it is worthing to note that VFAs molar proportions were significantly influenced by diets, with higher acetic and butyric acid concentrations in caecum of rabbits fed MS-Prob. Moreover, in this trial, probiotic assumption affected also caecal microbial composition and gut histomorphology. Compared to the control group, MS-Prob diet reduced harmful bacterial growth (*Escherichia coli*, *Bacillus* spp., *Clostridium* spp.) whereas significantly increased beneficial bacteria populations (*Lactobacillus* spp., *Bacteroides* spp.). Intestinal morphometric and morphologic evaluations (Table 4) stated a harmonic and improved functional tissue features in rabbit MS-Pro administered.

Gut microbiota exerts a multi-level role on digestive system. Through a modulation of metabolic, trophic and protective functions, microbiota aids the host in digestion of carbohydrates, proteins and lipids and produce various VFAs and other beneficial metabolites that in turn influence local microbial composition and provide to support gut mucosal integrity and host immune system [40]. Moreover, by reducing the pH or the oxygen in digestive tract, the subsequent modification of gut physico-chemical environment may antagonize the implantation of pathogenic bacteria. In this work, the MS-Prob was composed by *Streptococcus*, *Bifidobacterium* and *Lactobacillus* strains. Previous reports indicated that rabbit fed diets supplemented with *L*. *acidophilus* counted higher prevalence of caecal lactobacilli [24] and that *Lactobacillus* spp. exert a negative effect on cell proliferation and invasion capacity of harmful bacteria more than others bacteria [1]. In the present trial, probiotic strains manifested the ability to rise the beneficial microorganism populations (mainly *Lactobacillus* spp.) and to reduce potentially pathogenic bacteria like *Clostridium* and coliform in cecal digesta. These findings matched with those of Phuoc and Jamikorn [19] that showed an increase of the cecal lactobacilli population together with an increase of the cecal acetic acid concentration and a reduced intestinal coliform population in rabbits supplemented with *L*. *acidophilus*, alone or combined with *B*. *Subtilis*. An increment of lactobacilli, due to probiotic diets, was also reported by Khalifa et al [28] and Wlazło et al [41] who found an increased count of lactic acid bacteria as well as a decrease of coliforms in the intestinal tract of rabbit. Similarly, Helal et al [34] found an increment of *Lactobacillus* spp. and a reduction of *E*. *coli* and *C*. *perfringens* in rabbit fed probiotic complex containing *Saccharomyces cerevisiae* and *Bacillus subtilis*. In this study, rabbits fed MS-Prob diet had a greater acetic and butyric acid concentration in the caecal content than the rabbits fed control. These organic acids are metabolic end-products of microbiota carbohydrate fermentation; thus, these findings were indicative of an increased cecal microbial production and fermentation activity. To date, a greater number of intestinal lactobacilli enhances the hydrolytic enzymes activity resulting to an increase in caecal acetic acid concentration [24] and a decrease of intestinal coliform population, which consequently improves gut efficiency. Further, probiotic administration may raise the number of some caecal beneficial microorganism that are known to produce butyrate [1]. Butyric acid regulates the large intestine functions providing for the nutritional support and the integrity of intestinal villi, and influencing GCs differentiation and mucin composition which in turn improve the intestinal mucous barrier and the digestive functions [42].

Many studies have demonstrated that the selection of local microbiota and the modification of gut environment by probiotics have a direct influence on morphology, functions and mucus composition of the intestinal tract. Beneficial bacteria in probiotic formulations can influence villus architecture, promote intestinal cells turnover and differentiation, and improve the production of mucus and tight junction proteins. These changes enhance the functionality of gut tract optimizing nutrients’ uptake, therefore leading to improved production performance and overall health status in rabbits.

In this study, dietary probiotic administration modified the gut histomorphometric features; in particular, rabbit in MS-Prob group showed significantly increased duodenal VH, CD and a consequent increased VH:CD ratio (Table 5; Figures 1, 2). Lastly, microscopic analysis further confirmed the augmented duodenal absorptive surface area. Morphometric data of intestinal villi and crypts are important indicators of digestive well-being. An increase in VH may be directly linked with the extension of absorptive surface area and cell proliferation [43], while the CD rate may be related to the intensity of cells turnover, since crypt cells provide to renewal the absorptive epithelial cells that lining the intestinal wall. Hence, an elevated ratio of VH:CD indicates enhanced nutrient absorption capacity [16]. The villi elongation and the increased surface area could also improve digestion and absorption of nutrients enhancing the brush board enzyme expression and activity. In addition, intestinal crypts cells may be involved in protein digestibility through the synthesis of enzymes that control the production of pepsin [44]. The influence of probiotics on intestinal architecture may be explained by different mechanism. Lactobacillus spp. dietary supplementation was found to increase VH and CD by the production of VFAs which could promote cells proliferation in the intestinal villi and crypts [1,45]. On the other side, also the enhanced activity of digestive enzymes could improve the turnover rate of the intestinal epithelium in animals fed prebiotics [46]. Findings of the present study corroborated the results of histological evaluation reported by Nwachukwu et al [44] after the administration of *Lactobacillus acidophilus* that increased the VH, CD and VH:CD ratio in rabbits. Similarly, *S*. *Cerevisiae* and *Bacillus subtilis* or their mixture significantly improved the VH, CD and VH:CD ratio of experimental rabbit groups [34]. Interestingly, in growing rabbits, dietary probiotic supplementation administered at different concentration (0.05%, 0.1%, 0.15% and 0.2%) influenced the VH in a different way among the probiotic levels, with the highest values observed at 0.05% and 0.1%, and the lowest values at 0.15% and 0.2% [47]. While, El-Sawy et al [22] that dietary addition of different types of probiotics in rabbit increased the number of the villi, the mucosal glands, and the thickness of the muscularis externa and serosa layers in the caecum.

Regarding PAS-stained samples analyzed in the present investigation, it was showed a regular density of GCs, suggesting an improvement in production of mucus, since these cells influence the quantity and quality of the mucus that lining the villi. Otherwise, many studies reported that the dietary probiotic may also act on the mucus layer composition reinforcing the natural barrier against pathogenic bacteria and other injuries [33]. Moreover, a well-functioning intestinal barrier allows to a greater absorption of available nutrients, while avoid the ingestion of harmful substances. Moreover, the changes evidenced on intestinal mucosal morphology and microbiota composition of MS-Prob rabbit provide evidence of the positive effects of this mixture on the functionality and the integrity of gut.

## CONCLUSION

The dietary supplementation with the tested MS-Prob highlighted its beneficial effect on productive performance and the overall health status of rabbits. The enhancement of intestinal features and caecal microenvironment may be linked with an optimized nutrient uptake and absorptive capacity that led to an improvement of growth indices of rabbit fed probiotics. Furthermore, feeding rabbit with probiotics exerted a remarkable effect on antioxidative enzymes activity and lipid profile. Thus, these findings indicated that including probiotics may be considered a promising sustainable feeding strategy in rabbits.

## Figures and Tables

**Table 1:** Ingredients and chemical composition of the basal-diet fed to rabbits

Ingredients	Content (g/kg)
Dehydrated alfalfa meal	285
Dehydrated beet pulp	285
Corn	200
Soybean meal (48% CP)	100
Wheat middlings	84.5
Vitamin-mineral premix^1)^	50
Monocalcium phosphate	50
Sodium chloride	40
Calcium propionate	25
L-lysine	25
DL-methionine	25
Cane molasses	20
Yeast	10
Magnesium oxide	10
Magnesium carbonate	10
Chemical composition (g/kg as-fed)	
Dry matter	891
Crude protein	154
Ether extract	24
Crude fiber	141
Neutral-detergent fiber	268
Acid-detergent fiber	167
Lignin	39
Ash	69
Digestible energy (MJ/kg)	10.61

1) Provided per kg of diet: vitamin A 12,500 IU; vitamin D_3_ 1,500 IU; vitamin E 30 mg; vitamin B_1_ 1.5 mg; vitamin B_2_ 5 mg; vitamin B_6_ 2 mg; vitamin B_12_ 0.02 mg; vitamin PP 20 mg; vitamin K_3_ 2.5 mg; folic acid 0.75 mg; pantothenic acid 10 mg; D-biotin 0.1 mg; choline chloride 300 mg; MnSO_4_ 150 mg; FeSO_4_ 5 mg; ZnSO_3_ 75 mg; CuSO_4_ 5 mg; KI 1 mg; CoSO_4_ 0.2 mg; Na_2_SeO_3_ 0.1 mg.

CP, crude protein.

**Table 2:** Effect of multi-strain probiotic (MS-Prob) on growth performance, carcass yield and meat nutritional composition of rabbits

Item	Control	MS-Prob	Pooled SEM	p-value
Growth performance				
Initial live weight (g)^1)^	1,108	1,092	31.4	0.103
Final live weight (g)^1)^	2,105	2,202	59.9	0.004
Daily weight gain (g/d)^1)^	23.74	26.43	1.03	0.003
Feed intake (g/d)^2)^	105	108	0.47	0.055
Feed conversion ratio (g/g)^2)^	4.42	4.09	0.17	<0.001
Mortality (%)	20.0	10.0	0.14	<0.001
Carcass traits				
Slaughter weight (g)	2,060	2,153	90.45	0.038
Hot carcass weight (g)	1,240	1,333	52.05	0.032
Carcass yield (%)	60.19	61.91	1.13	0.025
Meat nutritional composition (%)				
Moisture	73.50	73.44	0.10	0.088
Protein	23.29	23.58	0.34	0.129
Lipids	1.74	1.59	0.29	0.093
Ash	1.47	1.39	0.12	0.113

1) Individual data.

2) Average pen data.

SEM, standard error of the mean.

**Table 3:** Effect of multi-strain probiotic (MS-Prob) on blood serum lipids and antioxidant capacity of rabbits

Item	Control	MS-Prob	Pooled SEM	p-value
Serum lipids (mg/dL)				
Cholesterol	157.44	121.53	0.88	0.001
LDL	161.01	153.32	0.57	0.002
HDL	61.12	55.75	0.48	0.003
Triglyceride	73.67	65.33	0.44	0.003
Antioxidant capacity				
GPx (U/mL)	16.75	27.89	2.75	0.002
GST (μmol/h/mL)	1.40	1.97	0.06	0.003
CAT (μmol H_2_O_2_)	61.03	81.89	3.98	0.001
SOD (U/mL)	3.01	4.77	0.79	0.003
TBARS (nmoL/mL)	0.31	0.18	0.05	0.001

SEM, standard error of the mean; LDL, low-density lipoprotein; HDL, high-density lipoprotein; GPx, glutathione peroxidase; GST, glutathione S-transferase; CAT, catalase; SOD, superoxide dismutase; TBARS, thiobarbituric acid-reactive substances.

**Table 4:** Effect of multi-strain probiotic (MS-Prob) on caecal characteristics and microflora population of rabbits

Item	Control	MS-Prob	Pooled SEM	p-value
Caecal characteristics				
pH	6.09	6.13	0.04	0.088
Ammonia-N (mmol/L)	3.78	4.04	1.09	0.351
Total VFA (mmol/L)	71.5	74.8	8.64	0.182
Acetic acid (mol/100 mol VFA)	81.4	84.4	1.97	0.047
Propionic acid (mol/100 mol VFA)	2.01	2.71	0.19	0.113
Butyric acid (mol/100 mol VFA)	11.5	14.9	0.47	0.019
Valeric acid (mol/100 mol VFA)	0.62	0.74	0.13	0.202
Caecal microbiota (log CFU/g)				
*Bacillus* spp.	6.29	5.01	0.49	0.003
*Bacteroides* spp.	3.03	4.63	0.52	0.004
*Clostridium* spp.	4.25	2.12	0.40	0.001
*Escherichia coli*	4.98	2.44	0.59	0.001
*Lactobacillus* spp.	2.16	4.01	0.41	0.001

SEM, standard error of the mean; VFA, volatile fatty acid; CFU, colony forming unit.

**Table 5:** Effect of multi-strain probiotic (MS-Prob) on duodenal histomorphometry of rabbits^1)^

Item	Control	MS-Prob	Pooled SEM	p-value
	
Villus height (μm)	678	761	29.8	0.001
Villus width (μm)	73.1	79.5	3.39	0.066
Crypt depth (μm)	124.8	181.0	3.95	0.002
Villus height/crypt depth	5.43	4.20	1.041	0.002
Villus surface area (mm^2^)	981	1,032	37.1	0.035

1) Each value represents the mean of ten rabbits per group.

SEM, standard error of the mean.
